# Author Correction: Impact of daily high dose oral vitamin D therapy on the inflammatory markers in patients with COVID 19 disease

**DOI:** 10.1038/s41598-021-97181-y

**Published:** 2021-08-30

**Authors:** Maheshwar Lakkireddy, Srikanth Goud Gadiga, R. D. Malathi, Madhu Latha Karra, I. S. S. V. Prasad Murthy Raju, Ragini, Sangeetha Chinapaka, K. S. S. Sai Baba, Manohar Kandakatla

**Affiliations:** 1 Department of Orthopaedics/ Biochemistry/ Internal Medicine, Nizam’s Institute of Medical Sciences, Punjagutta, Hyderabad, Telangana India; 2 Department of General Medicine/ Biochemistry, Gandhi Medical College, Secunderabad, Telangana India

Correction to: *Scientific Reports* 10.1038/s41598-021-90189-4, published online 20 May 2021

The original version of this Article contained errors.

In Table 4, the Variable order “LDH (U/l)”, “IL6 (pg/ml)” and “CRP (mg/l)” were incorrectly given as “CRP(mg/l)”, “LDH(U/l)” and “IL6(pg/ml)” respectively. The correct and incorrect Variable order appears below.

Incorrect:

**Table:** 

Variable	Pre (n = 15)		Post (n = 15)		Pre vs Post	
	Mean ± SD or median (IQR)	95% CI of mean/median	Mean ± SD or median (IQR)	95% CI of mean/median	t or z statistic	p value
CRP (mg/l)	385 ± 206^#^	271–499*	254 ± 84^#^	208–300*	− 3	0.007
LDH (U/l)	33 ± 35^#^	14–52*	3 ± 4^#^	1–5*	− 3	0.004
IL6 (pg/ml)	57 (22–96)	22–96	8 (3–16)	3–16	3	0.0003

Correct:

**Table:** 

Variable	Pre (n = 15)		Post (n = 15)		Pre vs Post	
	Mean ± SD or median (IQR)	95% CI of mean/median	Mean ± SD or median (IQR)	95% CI of mean/median	t or z statistic	p value
LDH (U/l)	385 ± 206^#^	271–499*	254 ± 84^#^	208–300*	− 3	0.007
IL 6 (pg/ml)	33 ± 35^#^	14–52*	3 ± 4^#^	1–5*	− 3	0.004
CRP (mg/l)	57 (22–96)	22–96	8 (3–16)	3–16	3	0.0003

Furthermore in Table 5, the Variable order “LDH (U/l)”, “IL6 (pg/ml)” and “CRP (mg/l)” was incorrectly given as “CRP(mg/l)”, “LDH(U/l)” and “IL6(pg/ml)” respectively. The “Post (n=15), 95% CI of mean/median” value for the Variable “CRP (mg/l)” was incorrect. The correct and incorrect Variable order and corresponding values appear below.

Incorrect:

**Table:** 

Variable	Pre (n = 15)		Post (n = 15)		Pre vs Post	
	Mean ± SD or median (IQR)	95% CI of mean/median	Mean ± SD or median (IQR)	95% CI of mean/median	t or z statistic	p value
CRP (mg/l)	257 ± 106^#^	198–316*	235 ± 88^#^	186–284*	− 0.9	0.4
LDH (U/l)	4 (2–9)	2–9	1 (0–9)	0–9	49	0.6
IL6 (pg/ml)	8 (2–19)	2–19	5 (2–8)	2–9	42	0.3

Correct:

**Table:** 

Variable	Pre (n = 15)		Post (n = 15)		Pre vs Post	
	Mean ± SD or median (IQR)	95% CI of mean/median	Mean ± SD or median (IQR)	95% CI of mean/median	t or z statistic	p value
LDH (U/l)	257 ± 106^#^	198–316*	235 ± 88^#^	186–284*	− 0.9	0.4
IL 6 (pg/ml)	4 (2–9)	2–9	1 (0–9)	0–9	49	0.6
CRP (mg/l)	8 (2–19)	2–19	5 (2–8)	2–8	42	0.3

The original Article has been corrected.

